# The Administration of Nitrous Oxide as a Preliminary to Ether Anæsthesia

**Published:** 1895-03

**Authors:** Bertram M. H. Rogers

**Affiliations:** Physician and Pathologist to the Bristol Hospital for Sick Children and Women


### THE ADMINISTRATION OF NITROUS OXIDE AS A PRELIMINARY TO ETHER ANÆSTHESIA.

BY

BERTRAM M. H. ROGERS, B.A., M.D., B.Ch. Oxon.,

*Physician and Pathologist to the Bristol Hospital for Sick Children and Women.*

As long ago as 1876, Clover described how before ether anæsthesia it was advisable to administer nitrous oxide; so that were it not for the neglect of this valuable suggestion, I should feel that an apology was required for bringing up such a hackneyed question. All the unpleasant sensations experienced during the early stages of ether anæsthesia may be avoided by giving a small quantity of nitrous oxide first.

First of all, I wish to express my regret that chloroform is still so largely used, when we have a far safer and an equally satisfactory anæsthetic in ether. As far as I can judge, the causes for its almost universal use—certainly in country practice—are, that its administration requires no expensive, more or less complicated apparatus, and that its use is far more economical. But when we bear in mind the not inconsiderable risk attendant on all cases of chloroform anæsthesia, I am convinced that its use should be restricted to those cases in which ether, for some particular reason, is to be discarded. There must be, in all cases of anæsthesia sufficiently profound for surgical operations, a certain amount of risk; but we may always feel when administering ether that when once sufficiently deep narcosis has been produced, timely warning will be given of any dangerous condition; whereas with chloroform, patients not infrequently pass to a more or less dangerous stage with great and unexpected suddenness. Whether or not we hold, with the Hyderabad Commission, that cardiac failure in chloroform anæsthesia is only secondary to respiratory failure matters little in comparing the relative safety of ether and chloroform, for there appears to be no serious risk of either when ether is given.

Without wishing to raise the whole question of the choice of anæsthetics, the best methods of administering them, and their relative safety or danger, I must say I believe that a large number of the fatalities from anæsthetics are due to inexperience, or perhaps primary want of knowledge, on the part of the anæsthetist; or want of attention during the operation to the patient's condition. Experience should be gained during the time of student life, and should not, as is the usual custom, be postponed till the qualifications to practise are obtained. Plenty of opportunities are given at the hospitals attached to medical schools for students to learn how to give anæsthetics, but they are much too often neglected for the superior attractions of the operation. Theoretical knowledge of administration, though not so useful, is of no mean value; but few of our medical schools include a course of instruction in anæsthetics in their syllabus, and I have never heard of a question on the subject being asked in any examination. The anæsthetist should be wholly engaged in giving the anæsthetic, and should pay no more attention to the operation and doings of the surgeon than to see that his patient is sufficiently under to render the operation possible without movement or pain.

Now to the point to which I wish specially to call attention. Those who have had the misfortune to have taken ether will know that the inhalation of the vapour is not altogether pleasing.

The odour is pungent, and the taste burning and most unpleasant; and it is impossible, even when given by the most skilled hands, to avoid these disagreeable sensations. When, however, nitrous oxide is given in the manner I shall presently mention, all this unpleasantness is lost: further, there is neither struggling nor cough; in fact, the patient passes from the far from unpleasant sensations of nitrous oxide anæsthesia to ether narcosis quite insensibly and quietly. I have had nitrous oxide administered to me more than once, and can assure those who have never experienced it that, beyond a peculiar sweet taste and a tendency to take deeper respirations than usual, the sensation is on the whole pleasurable. In administering gas before ether, only sufficient of the former anæsthetic is given to render the patient partially insensible—only till the sensation of lightness of the head is induced; and though I am not prepared to say that any anæsthesia of the air passages is produced, practical experience teaches that after a few—six or eight—respirations the irritating effect of the ether on the mucous membrane of the respiratory tract is not observed. On the last occasion on which I took nitrous oxide I attempted to count the number of respirations I took before I became insensible, and on recovery found I only remembered taking twelve. No doubt I should have felt the pain of the tooth extraction if it had taken place then; but this shows how soon loss of consciousness comes on.

The apparatus I use is Hewitt's modification of Clover's ether inhaler: others are in use, but I find this answers its purpose well, and I shall continue to use it till one evidently superior is introduced. The gas-bag, previously filled with nitrous oxide, is fitted on to the dome which has been warmed and supplied with ether. All the fittings of the apparatus should be so accurately made that there is no leakage of gas or ether; so that when the gas is turned on, no taste of ether vapour is noticed. The valves are so arranged that respiration of air alone is allowed at first, by which the patient gets accustomed to the face-piece and can be induced to breathe properly—a thing often not done at first,—and the anæsthetist can hear that the valves are working properly. After a few respirations the gas is turned on, and is drawn from the bag to the patient's lungs and expired through the valves. About six respirations in this way are permitted, when the valve arrangement is turned off, so that respirations now pass from and to the bag, and at the same time a small turn is given to the ether dome. In a short time signs of slight nitrous oxide narcosis are noticed, but should not be allowed to proceed too far. There should not be much lividity; but should it become marked, a single breath of air causes it to disappear. Meanwhile the ether has been turned on till now it is nearly at full, and in a short time the patient is ready. The great difficulty to avoid is, allowing the patient to come round between the gas and ether narcosis—a thing very easily done if too much air is admitted. Only experience can teach the anæsthetist how much, and when, air should be given. Deep nitrous oxide anæsthesia is not desired or required. It will be found that two gallons of gas is about the amount required; but each patient has a personal equation both for nitrous oxide and for ether, and the anæsthetist has to find it out by careful and cautious observation. I believe by this method of administration after-vomiting is less frequent. But much, too, depends on keeping the patient equably under the anæsthetic at the time of the operation. There should be deep anæsthesia at first, especially if the operation is on a sensitive part, but much lighter anæsthesia is sufficient subsequently; but it must be maintained equably, or as equably as is possible.

To those who have no experience of this method, I can confidently recommend it as by far the simplest both for the patient and the administrator; and if comfort is concerned, I can unhesitatingly say that it is the best yet devised.

---

### THE BLOOD-ALTERATIONS OF ETHER-ANÆSTHESIA.

Dr. J. C. Da Costa (*Medical News*, March 2, 1895) considers that in ether-anæsthesia the cause of the anæsthetic state is not merely the direct action of ether upon the nerve-elements, but that it involves likewise an alteration in the composition of the blood. He says that diminution in hæmoglobin is constant after the inhalation of ether, that ether-pneumonia may possibly be due to the action of intense cold upon the lungs, produced by the action of ether-vapour, that prolonged anæsthesia profoundly deteriorates the blood and strongly militates against recovery, and that hence rapidity of operation is most desirable.

